# Congenital thrombotic thrombocytopenic purpura and human leukocyte antigen analysis–an amazing clue

**DOI:** 10.1016/j.htct.2025.103746

**Published:** 2025-02-15

**Authors:** Cevat İlteriş Kıkılı, Demet Kıvanç, Hayriye Şentürk Çiftçi, Mustafa M. Özbalak, Mustafa N. Yenerel, Meliha Nalçacı, Fatma S. Oğuz, Sevgi K. Beşışık

**Affiliations:** Department of Internal Medicine, Istanbul University, Istanbul Faculty of Medicine, Istanbul, Turkey

Dear Editor,

Thrombotic thrombocytopenic purpura (TTP) is an extremely rare thrombotic microangiopathy characterized by ADAMTS-13 deficiency. Deficiency of ADAMTS-13, a cleaving protease of von Willebrand Factor (vWF), leads to the formation of ultra-large vWF multimers. When these multimers interact with platelets, platelet-rich thrombi form in microvascular vessels leading to organ ischemia. If not treated promptly and properly, TTP can be fatal. Congenital TTP, caused by a mutation in the gene encoding ADAMTS-13, is very rare accounting for about 10 % of TTP patients. >150 mutations of this gene have been identified so far. Acquired TTP, an autoimmune form of TTP which comprises approximately 90 % of all cases, is caused by antibodies against ADAMTS-13.^1^, ^2^, ^3^, ^4^, ^5^

The human leukocyte antigen (HLA) region, with >200 genes, most of which are related to the immune system, is located at position 6p21.3 on the short arm of chromosome 6. Class II HLA molecules play a crucial role in antigen presentation; their association with autoimmune diseases has been reported in the literature.^6^

Studies evaluating the association between acquired TTP and HLA report that some HLA alleles predispose to TTP whereas others are protective. Particularly in individuals of European ancestry, HLA-DRB1×11 was found to be a predisposing factor, whereas HLA-DRB1×04 was found to be protective. Considering the autoimmune nature of acquired TTP and the role of HLA class II molecules in antigen presentation and autoimmune diseases, the association between acquired TTP and HLA cannot be denied.^7^, ^8^, ^9^, ^10^

According to the hypothesis for acquired TTP described in the literature, the role of HLA is to determine which peptide derived from the CUB-2 domain of ADAMTS-13 will be presented by major histocompatibility complex (MHC) class II genes to CD4^+^ *T* cells. HLA genes encode the proteins that form the peptide-binding region in MHC class II molecules. Immune tolerance to ADAMTS-13 is lost in the presence of HLA molecules which encode peptide-binding regions that enable the presentation of high-affinity antigenic peptides. This leads to the formation of autoantibodies against ADAMTS-13 resulting in TTP.^7^^,^^8^

To the best of our knowledge there are no published studies investigating an association between HLA and congenital TTP characterized by a mutation in the gene encoding ADAMTS-13. This study aimed to contribute by analyzing HLA class II proteins in five patients diagnosed with congenital TTP and to reveal a new clue for the elucidation of the pathogenesis of congenital TTP.

## Patients and methods

Five patients diagnosed with congenital TTP based on clinical and laboratory data who were followed up at Istanbul University, Istanbul Medical Faculty, Department of Hematology, were enrolled in this study. Verbal and written informed consent was obtained from the participants. This study was conducted in accordance with the Declaration of Helsinki with the approval of the Istanbul Medical Faculty Clinical Research Ethics Committee (numbered 807226 - 31/03/2022).

A control group, followed up at the institution's Tissue Typing Laboratory in the Department of Medical Biology, consisted of 150 healthy individuals with similar ages and genders to the patient group.

Blood was collected in 10 mL ethylenediaminetetraacetic acid (EDTA) tubes and DNA was extracted. Subsequently, HLA class 2 (HLA-DRB1 and HLA-DQB1) typing was performed as low resolution (2 digits) using sequence-specific oligonucleotide probe (SSOP) polymerase chain reaction (PCR - HLA luminescence and PCR-Hybridization methods). Then, the five most common HLA-DRB1 and three prevalent HLA-DQB1 alleles were identified and high-resolution (4-digit) typing was performed using polymerase chain reaction with sequence-specific primers (PCR-SSP). The LIFECODES HLA-DRB1 rapid typing kit (Immucor GTI 628923S, USA), and HLA-DQA1/B1 combi typing kit (Immucor GTI 628930, USA) were used for SSOP-PCR. HLA-DRB1 and HLA-DQB1 typing kits (QIAGEN, USA) were used for PCR-SSP.

## Results and discussion

Table 1 shows the *HLA-DRB1* and *HLA-DQB1* alleles. In addition, commonly inherited allele pairs (*HLA-DRB1×11* + *HLA-DQB1×03, HLA-DRB1×15* + *HLA-DQB1×06, HLA-DRB1×07* + *HLA-DQB1×02, HLA-DRB1×13* + *HLA-DQB1×06, HLA-DRB1×08* + *HLA-DQB1×06*) of the five congenital TTP patients are shown in Table 2.

**Table 1:** Five most common *HLA-DRB1* and three prevalent *HLA-DQB1* alleles.

HLA-DRB1	n	%	HLA-DQB1	n	%
HLA-DRB1×11	4	40.0	HLA-DQB1×03	7	70.0
HLA-DRB1×04	2	20.0	HLA-DQB1×06	2	20.0
HLA-DRB1×13	2	20.0	HLA-DQB1×05	1	10.0
HLA-DRB1×15	1	10.0			
HLA-DRB1×01	1	10.0			
**HLA-DRB1**	**n**	**%**	**HLA-DQB1**	**n**	**%**
HLA-DRB1×11:01	2	20.0	HLA-DQB1×03:01	5	50.0
HLA-DRB1×11:04	2	20.0	HLA-DQB1×03:02	2	20.0
HLA-DRB1×01:02	1	10.0	HLA-DQB1×05:01	1	10.0
HLA-DRB1×04:02	1	10.0	HLA-DQB1×06:03	1	10.0
HLA-DRB1×04:05	1	10.0	HLA-DQB1×06:04	1	10.0
HLA-DRB1×13:01	1	10.0			
HLA-DRB1×13:02	1	10.0			
HLA-DRB1×15:01	1	10.0

**Table 2:** Percentages and frequencies of HLA-DRB1 + HLA-DQB1 allele pairs frequently reported in congenital TTP.

Allele pairs	n	%
HLA-DRB1×11 + HLA-DQB1×03	4	80
HLA-DRB1×15 + HLA-DQB1×06	1	20
HLA-DRB1×07 + HLA-DQB1×02	0	0.0
HLA-DRB1×13 + HLA-DQB1×06	2	40
HLA-DRB1×08 + HLA-DQB1×06	0	0.0

Considering the five congenital TTP patients in this study, the HLA-DQB1×03:01 allele was found in all patients and the HLA-DQB1×03 allele was found in 70 % of the patients, whereas it was detected in only 42.7 % of the control group. The HLA-DRB1×11 allele was found in 40 % of the patients while it was detected in only 21.3 % of the control group. The HLA-DQB1×03:01 allele was detected in 50 % of the patients, whereas it was detected in 27 % of the control group. In addition, the HLA-DRB1×11 + HLA-DQB1×03 allele pair was detected in four of the five congenital TTP patients (80.0 %) and only 34.66 % of the control group.

Consequently, in this study, the HLA-DRB1×11 + HLA-DQB1×03 allele pair, HLA-DQB1×03, HLA-DRB1×11 and especially HLA-DQB1×03:01 were found to be more frequent in patients with congenital TTP compared to healthy controls, similar to results for acquired TTP reported in the literature. However, further studies are needed because of the small sample size due to the rarity of the disease. In addition, two of the five patients with congenital TTP were siblings, suggesting that shared HLAs may be inherited from their parents.^9^, ^10^

In the literature, there is a lack of information associating congenital TTP with the HLA system. In contrast to acquired TTP, the role of HLA in the pathogenesis of congenital TTP remains unclear. To the best of our knowledge this is the first-ever HLA analysis of congenital TTP, providing valuable insights into its pathogenesis and the role of HLA in affected patients.

## Informed consent

Informed consent was obtained from all individual participants included in the study.

## Consent for publication

Consent for publication was obtained for every individual person's data included in the study.

## Funding

This study was not supported by any funding.

## Ethical approval

All procedures performed in studies involving human participants were in accordance with the ethical standards of the institutional and/or national research committee and with the 1964 Helsinki declaration and its later amendments or comparable ethical standards. This study was conducted in accordance with the Declaration of Helsinki with the approval of the Istanbul Medical Faculty Clinical Research Ethics Committee dated 31.03.2022 and numbered 807226.

## Author contributions

CİK: Investigation, Visualization, Writing – original draft; DKİ: Investigation, Visualization; HŞ: Investigation, Visualization; MMÖ, MNY and MN: Investigation, Writing – Review & editing; FSO: Investigation, Visualization, Writing – review & editing; SKB: Conceptualization, Project administration, Funding acquisition, Investigation, Supervision, Visualization, Writing – review & editing.

## Conflicts of interest

The authors declare that they have no conflict of interest.
